# CDKN2B gene rs1063192 polymorphism decreases the risk of glaucoma

**DOI:** 10.18632/oncotarget.15504

**Published:** 2017-02-18

**Authors:** Zhenxian Hu, Chenliang He

**Affiliations:** ^1^ Department of Ophthalmology, Tongde Hospital of Zhejiang Province, Hangzhou 310012, Zhejiang, China

**Keywords:** CDKN2B, single nucleotide polymorphism, glaucoma, meta-analysis, rs1063192

## Abstract

The aim of this meta-analysis was to evaluate the association between cyclin-dependent kinase Inhibitor-2B (CDKN2B) gene rs1063192 polymorphism and glaucoma risk. We searched the databases of PubMed, and Embase. Pooled odds ratios (ORs) and 95% confidence intervals (CIs) were calculated by using fixed-effect or random-effect models. A total of 14 case-control studies involving 11,316 cases and 24,055 controls were included. Meta-analysis showed that CDKN2B gene rs1063192 polymorphism was associated with a decreased risk of glaucoma. Stratification analysis of ethnicity indicated that rs1063192 polymorphism decreased the risk of glaucoma among Caucasians and Asians. Stratification analysis by type of glaucoma revealed that rs1063192 polymorphism conferred a protective factor of primary open-angle glaucoma (POAG) and non-POAG. Stratification by source of controls uncovered an association between rs1063192 polymorphism and glaucoma in groups of population-based controls. In conclusion, this meta-analysis indicates that CDKN2B gene rs1063192 polymorphism is significantly associated with a decreased risk of glaucoma.

## INTRODUCTION

Glaucoma is the second-leading cause of irreversible blindness in the world [1]. Data has been estimated that over 11.1 million people will be bilaterally blind from primary glaucoma by 2020 [1]. Glaucoma is characterized by degeneration of the optic nerve, and is usually associated with increased intraocular pressure [2]. The main types of glaucoma include primary open-angle glaucoma (POAG) and primary angle closure glaucoma (PACG). To date, the main pathogenesis for disease progression in glaucoma is still poorly understood. Studies suggested that various risk factors of glaucoma included age, elevated intraocular pressure (IOP), variable susceptibility of the optic nerve, vascular factors, diabetes, myopia, a positive family history and cigarette smoking [3]. Genetic factors also have been reported to play a vital role in the pathogenesis of glaucoma [4, 5]. Previous linkage-based studies demonstrated several genes were associated with glaucoma, including myocilin, CYP1B1, optineurin, and WDR36 [6–8]. Recent genome-wide association studies (GWASs) have identified several genetic variants associated with glaucoma [9], including cyclin-dependent kinase Inhibitor-2B (CDKN2B) gene.

CDKN2B has been identified to play an important role in G1 progression of the cell cycle [10]. Animal studies demonstrated that elevated IOP is associated with overexpression of CDKN2B, which leads to disruption in cell cycle causing abnormal cell proliferation [11]. GWAS studies for glaucoma showed direct association of CDKN2B region of 9p21 locus with glaucoma, identifying its significance as a risk factor [12, 13]. A host of studies [12–26] investigated the association between CDKN2B gene rs1063192 polymorphism and glaucoma susceptibility, but with conflicting findings. However, these studies were conflicting and inconclusive due to clinical heterogeneity, different ethnic populations, and small sample sizes. Therefore, we performed a comprehensive meta-analysis to clarify the possible association between CDKN2B gene rs1063192 polymorphism and glaucoma risk.

## RESULTS

### Characteristics of the included studies

**Figure 1:**
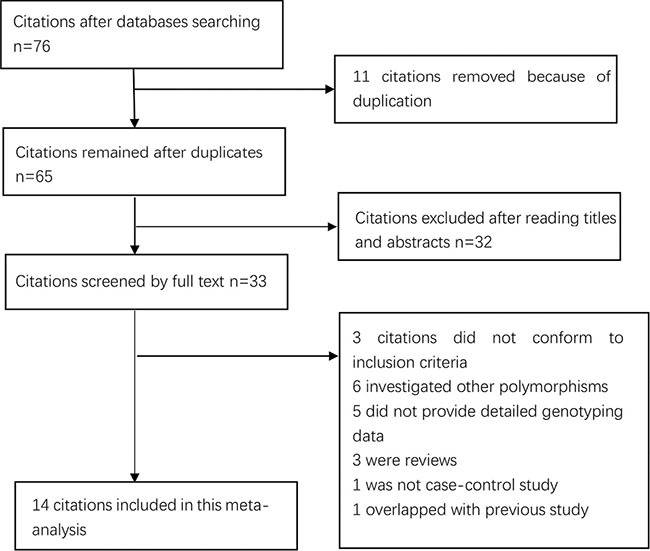
Selection for eligible publications included in this meta-analysis

We yielded a total of 76 citations the by searches of the PubMed and Embase databases and manual searching. 43 citations were removed after removing duplicates and screening the titles and abstracts. 33 citations were selected for further full text review. 19 citations (3 citations did not conform to the inclusion criteria; 6 investigated other polymorphisms; 5 did not provide detailed genotyping data; 1 overlapped with previous study; 3 were reviews; and 1 was not case-control study) were excluded after serious review. 14 eligible studies [12–17, 19–26] (11,316 cases and 24,055 controls) in this meta-analysis finally. Selection for eligible studies included in this meta-analysis was presented in Figure 1. The characteristics of the individual studies included in the meta-analysis are summarized in Table 1. The Newcastle-Ottawa Scale (NOS) scores of all included studies ranged from 6 to 7 stars, suggesting that these studies were of high methodological quality.

**Table 1:** Characteristics of included studies

Author and year	SOC	Country	Genotype methods	Ethnicity	Case			Control			HWE	NOS
					TT	TC	CC	TT	TC	CC		
Abu-Amero2016	HB	Saudi Arabia	PCR	Caucasian	53	32	2	58	31	5	Y	6
Ng2016	PB	Australia	Unclear	Caucasian	941	1022	278	1004	1564	608	Y	7
Burdon2015	PB	Australia	PCR	Caucasian	28	31	8	579	950	390	Y	7
Chen2015	HB	China	Unclear	Asian	770	348	39	592	303	39	Y	7
Williams2015	HB	South Africa	PCR	African	215	0	0	214	0	0	N	6
Philomenadin2015	HB	India	PCR	Asian	56	35	6	220	131	20	Y	6
Mabuchi2015	HB	Japan	PCR	Asian	301	113	11	115	66	10	Y	7
Micheal2014	HB	Pakistan	PCR	Asian	270	206	37	127	89	17	Y	7
Liu2013	PB	USA	Unclear	African	980	163	7	875	120	4	Y	7
Liu2013	PB	USA	Unclear	African	483	0	0	591	2	0	N	6
Cao2012	PB	USA	PCR	African	251	21	0	135	29	1	Y	7
Osman2012	PB	Japan	PCR	Asian	962	391	40	4155	2156	280	Y	6
Osman2012	PB	Japan	PCR	Asian	1267	486	47	4452	2425	330	Y	7
Dimasi2012	PB	Australia	Unclear	Caucasian	348	408	120	274	430	169	Y	7
Takamoto2012	HB	Japan	PCR	Asian	109	38	4	120	60	7	Y	7
Fan2011	PB	Netherland	TaqMan	Caucasian	170	172	47	102	155	51	Y	6

SOC, source of controls; PB, population-based controls; HB, hospital-based controls; HWE, Hardy–Weinberg Equilibrium; NOS, Newcastle-Ottawa Scale.

### Meta-analysis of CDKN2B gene rs1063192 polymorphism with glaucoma risk

**Figure 2:**
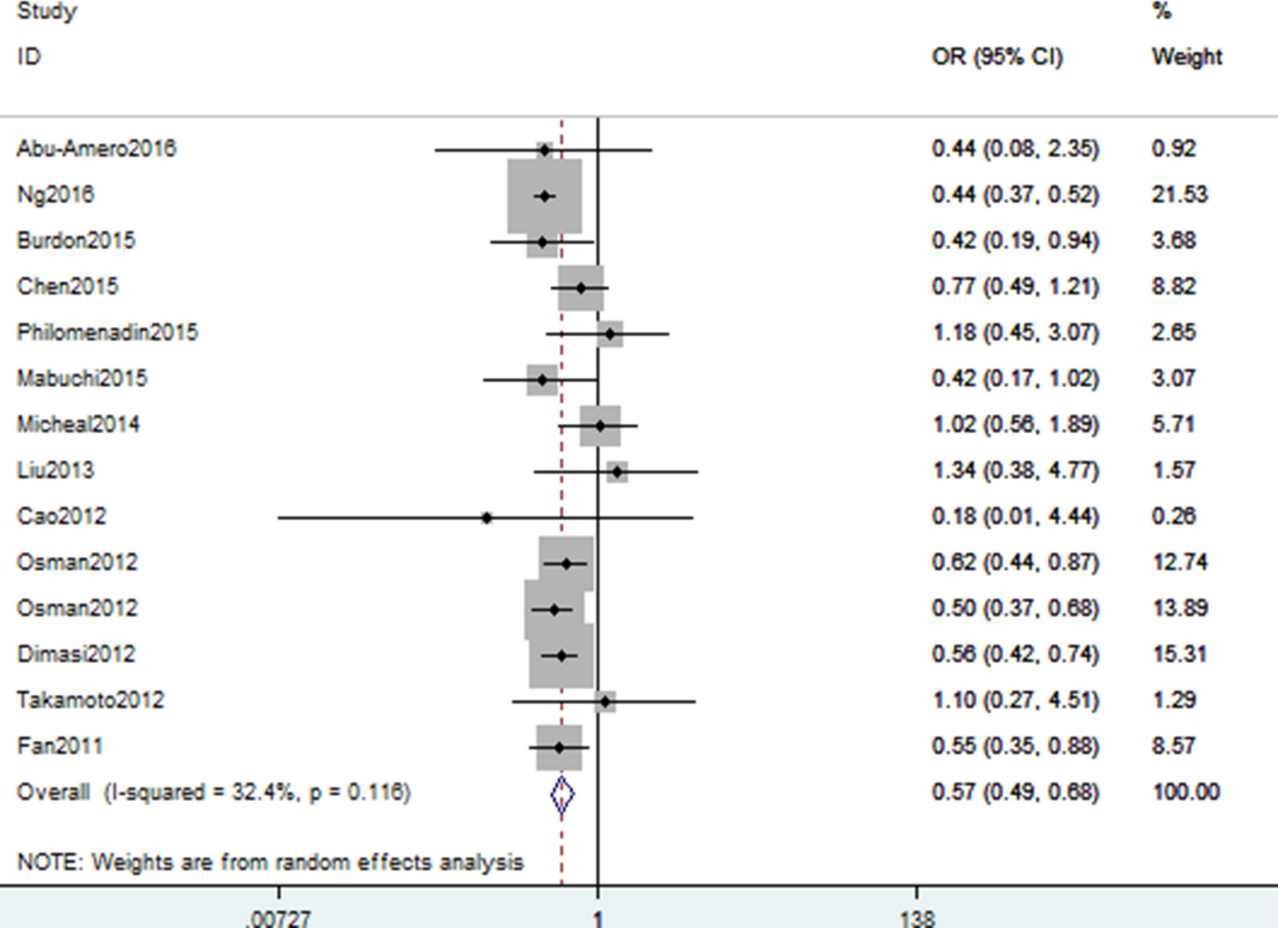
Forest plot shows odds ratio for the associations between rs1063192 polymorphism and glaucoma risk (CC vs. TT)

**Figure 3:**
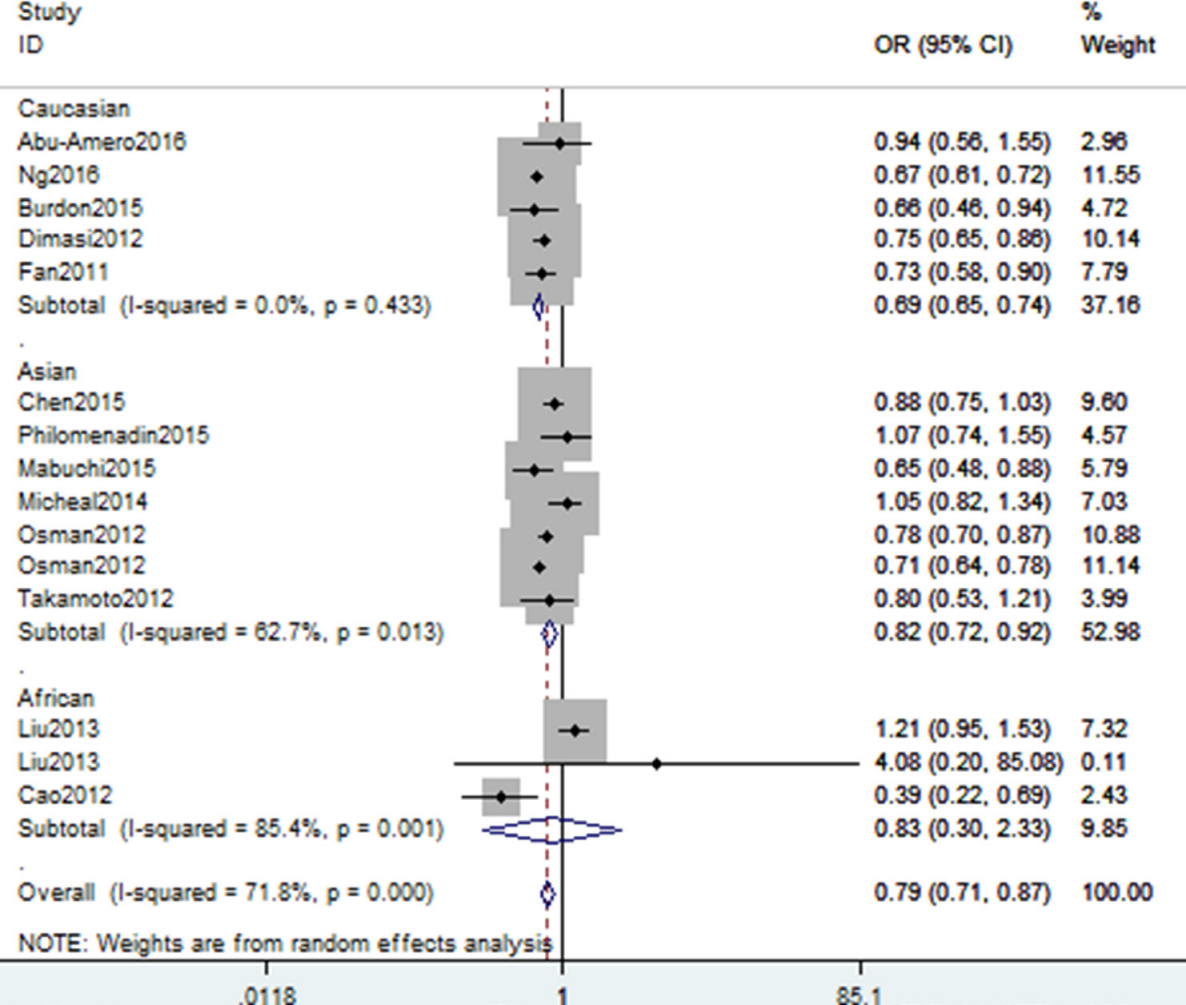
Stratification analysis of ethnicity shows odds ratio for the association between rs1063192 polymorphism and glaucoma risk (C vs. T)

**Figure 4:**
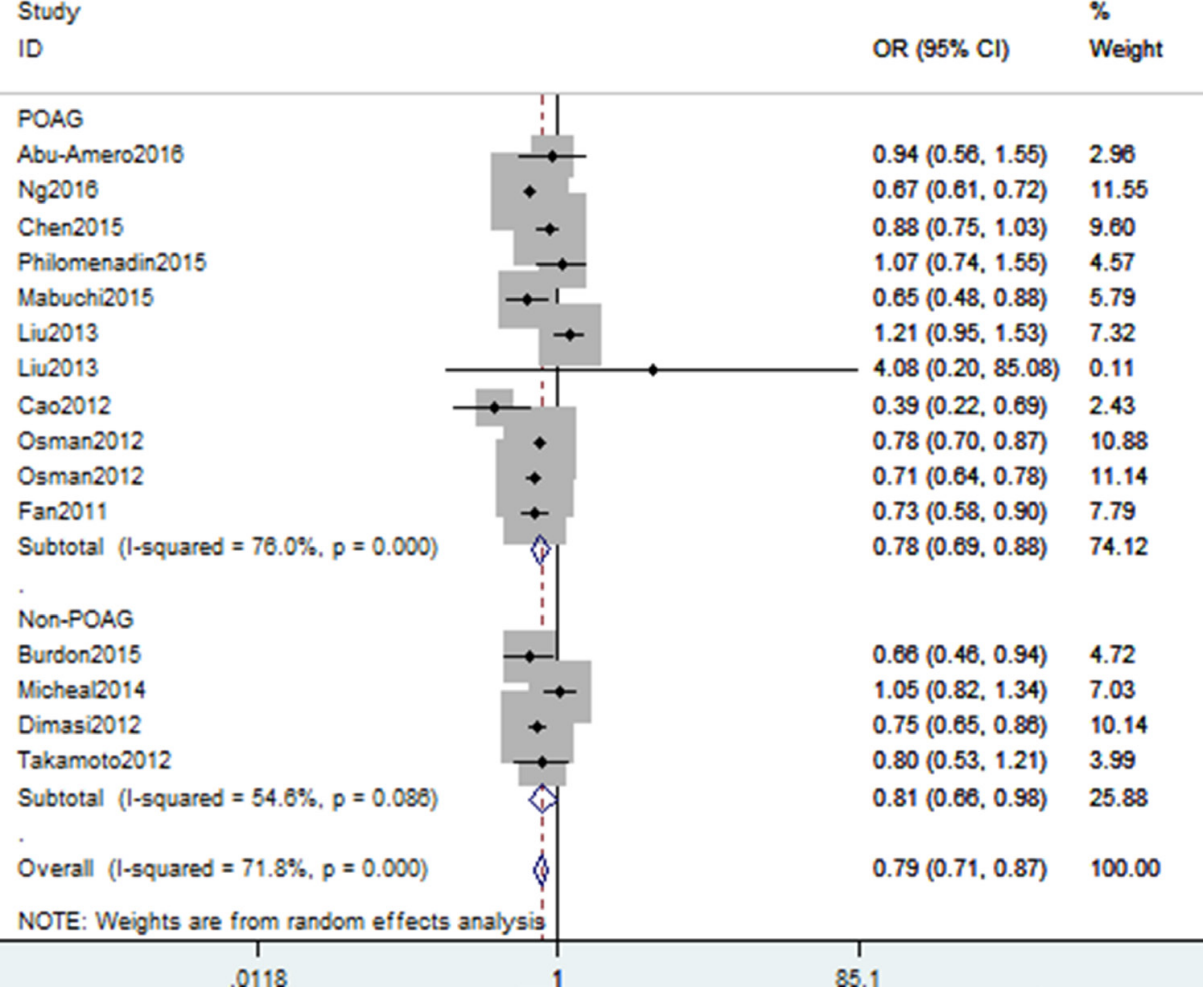
Stratification analysis of type of glaucoma between rs1063192 polymorphism and glaucoma risk (TT vs. CT + CC)

A summary of association between CDKN2B gene rs1063192 polymorphism with glaucoma risk is provided in Tables 2 and 3. In the overall analysis, we detected a significant association between CDKN2B gene rs1063192 polymorphism with the decreased risk of glaucoma (CC vs. TT: OR, 0.55; 95% CI, 0.49–0.61, *P* < 0.001, Figure 2). Stratification by ethnicity indicated that rs1063192 polymorphism was also significantly associated with a decreased risk of glaucoma among Caucasians and Asians (C vs. T, Figure 3). Similar result was also replicated among Africans in the allele model (C vs. T, Figure 3). Stratification analysis by type of glaucoma suggested that rs1063192 polymorphism was a protective factor of POAG and non-POAG (TT vs. CT + CC, Figure 4). Stratification by source of controls (SOCs) revealed an association between rs1063192 polymorphism and glaucoma in groups of population-based controls.

**Table 2:** Meta-analysis of association between *CDKN2B* rs1063192 polymorphism and the risk of glaucoma

Comparison	OR(95%CI)	*P*-value	*P* for heterogeneity	I^2^ (%)	Model
C vs. T	0.79 (0.72,0.87)	< 0.001	< 0.001	68.2	Random
CC + TC vs. TT	0.76 (0.68,0.85)	< 0.001	< 0.001	69.2	Random
CC vs. TT + TC	0.64 (0.58,0.71)	< 0.001	0.730	0.0	Fixed
CC vs. TT	0.55 (0.49,0.61)	< 0.001	0.286	15.3	Fixed
TC vs. TT	0.94 (0.82,1.07)	< 0.001	0.002	59.3	Random

* Bold values are statistically significant (*P* < 0.05).

**Table 3:** Summary of the subgroup analyses in this meta-analysis

Comparison	Category	Category	Studies	OR (95% CI)	*P*-value	*P* for heterogeneity
C vs. T	Ethnicity	Caucasian	5	0.71 (0.67,0.72)	< 0.001	0.724
		Asian	7	0.81 (0.72,0.91)	< 0.001	0.013
		African	3	0.79 (0.72,0.87)	0.404	0.001
	Type of glaucoma	POAG	12	0.78 (0.70,0.87)	< 0.001	< 0.001
		Non-POAG	3	0.81 (0.63,1.04)	0.096	0.037
	SOC	HB	6	0.88 (0.75,1.02)	0.080	0.174
		PB	9	0.75 (0.67,0.83)	< 0.001	0.001
CC + TC vs. TT	Ethnicity	Caucasian	5	0.66 (0.60,0.72)	< 0.001	0.575
		Asian	7	0.79 (0.69,0.90)	< 0.001	0.026
		African	3	0.63 (0.22,1.84)	0.402	0.001
	Type of glaucoma	POAG	12	0.75 (0.66,0.86)	< 0.001	< 0.001
		Non-POAG	3	0.78 (0.56,1.09)	0.143	0.038
	SOC	HB	6	0.87 (0.73,1.03)	< 0.001	0.204
		PB	9	0.71 (0.62,0.82)	0.107	< 0.001
CC vs.TC + TT	Ethnicity	Caucasian	5	0.62 (0.54,0.70)	< 0.001	0.898
		Asian	7	0.67 (0.56,0.80)	< 0.001	0.493
		African	2	1.12 (0.38,3.34)	0.838	0.247
	Type of glaucoma	POAG	11	0.63 (0.56,0.70)	< 0.001	0.719
		Non-POAG	3	0.69 (0.55,0.86)	0.001	0.375
	SOC	HB	6	0.80 (0.59,1.08)	0.148	0.701
		PB	8	0.62 (0.55,0.69)	< 0.001	0.779
CC vs.TT	Ethnicity	Caucasian	5	0.51 (0.44,0.58)	< 0.001	0.907
		Asian	7	0.62 (0.51,0.74)	< 0.001	0.258
		African	2	1.13 (0.38,3.35)	0.831	0.216
	Type of glaucoma	POAG	11	0.55 (0.48,0.63)	< 0.001	0.371
		Non-POAG	3	0.63 (0.41,0.96)	0.033	0.142
	SOC	HB	6	0.78 (0.58,1.06)	0.108	0.560
		PB	8	0.52 (0.47,0.59)	< 0.001	0.581
TC vs.TT	Ethnicity	Caucasian	5	0.71 (0.65,0.78)	< 0.001	0.615
		Asian	7	0.80 (0.71,0.90)	< 0.001	0.081
		African	3	0.64 (0.23,1.81)	0.404	0.002
	Type of glaucoma	POAG	12	0.78 (0.69,0.88)	< 0.001	0.002
		Non-POAG	3	0.83 (0.63,1.10)	0.190	0.122
	SOC	HB	6	0.89 (0.76,1.04)	0.138	0.294
		PB	9	0.75 (0.66,0.85)	< 0.001	0.004

SOC, source of control; PB, population-based controls; HB, hospital-based controls; POAG: Primary Open-Angle Glaucoma.

**Figure 5:**
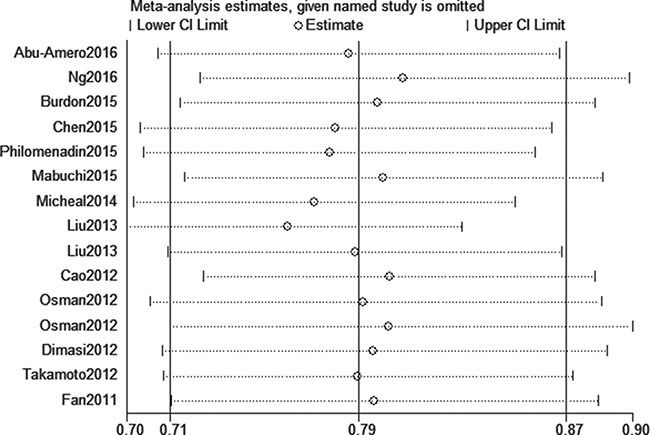
Sensitivity analysis for rs1063192 polymorphism and glaucoma risk (C vs. T)

**Figure 6:**
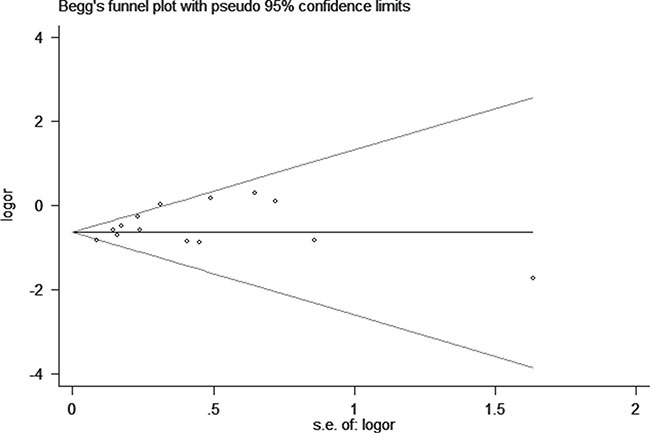
Begg's tests between rs1063192 polymorphism and glaucoma risk (CC vs. TT)

We assessed sensitivity by omitting each study once at a time in every genetic model for rs1063192 polymorphism. The pooled ORs for the effects of the single nucleotide polymorphism (SNP) on the risk for glaucoma risk indicated that our data was stable and trustworthy(C vs. T, Figure 5). Both Egger's and Begg's tests (CC vs. TT, Figure 6) were used to evaluated the publication bias of this meta-analysis. Our data showed no evidence of publication bias affecting the meta-analysis results. (data not shown).

## DISCUSSION

In this meta-analysis, our data indicated that CDKN2B gene rs1063192 polymorphism with the decreased risk of glaucoma. Stratification analysis by ethnicity found that rs1063192 polymorphism decreased the risk of glaucoma among Caucasians, Asians and Africans. Stratification analysis by type of glaucoma revealed that rs1063192 polymorphism was associated with a decreased risk of POAG and non-POAG.

Genes at the 9p21 locus, including CDKN2B, are strong candidates for POAG risk. CDKN2B gene plays a vital role in cell cycle, development and function of ophthalmic disorders [27]. Several GWASs among different ethnic groups provided strong evidence that gene variants at the CDKN2B locus of 9p21 are a crucial risk factor in the development of POAG [12, 23, 28, 29]. Recently, a host of studies [12–26] explored the association between CDKN2B gene rs1063192 polymorphism and glaucoma risk. However, these studies obtained conflicting results. The limitations of these studies [12–26] including clinical heterogeneity, different ethnic populations, and small sample sizes may contribute to the disaccords. Although association studies provide a powerful means of identifying genetic factors underlying glaucoma, most reported association studies lack sufficient statistical power. In such cases, meta-analysis can be used as an alternative. Therefore, we conducted this comprehensive meta-analysis to demonstrate the association between CDKN2B gene rs1063192 polymorphism and glaucoma susceptibility. To the best of our knowledge, this is the first meta-analysis to explore the relationship between this SNP and glaucoma risk.

In this meta-analysis, we combined data from included studies to evaluate genetic association between CDKN2B gene rs1063192 polymorphism and glaucoma. Our results showed a significant association between this SNP and glaucoma. Meta-analysis showed a significant association between rs1063192 polymorphism and glaucoma risk in Caucasians, Asians, and Africans, indicating that there was no significant ethnic differences about this SNP. It is of note that we only found the positive finding among Africans in the allele model, but the other four genetic models failed to replicate this association. The result of stratification analysis among Africans should be interpreted with caution, because the racial stratification of CDKN2B gene rs1063192 polymorphism and glaucoma regarding Africans included only three studies, which is insufficient to provide conclusive evidence. Thus, further studies using larger numbers of subjects are required. As for the subgroup analysis by type of glaucoma, we found rs1063192 polymorphism was associated with a decreased risk of POAG and non-POAG patients. Although the clinical heterogeneity of POAG and non-POAG is evident, meta-analysis did not find any different results between them. It is noteworthy that this meta-analysis only included 3 studies with limited sample size for non-POAG. Hence, we could exclude the likelihood of false-positive associations.

We believe our meta-analysis has some strengths. One, we included 14 studies with 11,316 cases and 24,055 controls in this study and the sample size of this meta-analysis was large. Two, sensitivity analysis provided evidence that our data about CDKN2B gene rs1063192 polymorphism were trustworthy and robust. Three, the power analysis indicated that our study had a power of 99.0% to detect the effect of rs1063192 polymorphism on glaucoma susceptibility, assuming an OR of 0.76.

However, this meta-analysis has some limitations that require further consideration. First, due to limited data, we could not perform further stratification analyses of other potential factors, such as age and gender. Second, our results were based on unadjusted estimates for confounding factors, which might have affected the final conclusions. Third, we could not assess potential gene-gene and gene-environment interactions because of the lack of relevant data. Fourth, the ethnicity-specific meta-analysis included data from Caucasians, Asians, and Africans, and thus, our findings are only applicable to these ethnic groups. Fifth, some unpublished studies may have been missed, although our data indicated no evident publication bias. Sixth, CDKN2B gene rs1063192 polymorphism would be not enough to explain the associations between CDKN2B gene and glaucoma risk. Seventh, the heterogeneity of this meta-analysis was high in some genetic models. Last but not least, the sample size of stratification analysis was limited in some ethnicities.

In conclusion, this meta-analysis demonstrates that CDKN2B gene rs1063192 polymorphism is significantly associated with a decreased risk of glaucoma. Larger scale studies of populations with different ethnicities are necessary to explore the roles played by this SNP of CDKN2B gene during the pathogenesis of glaucoma.

## MATERIALS AND METHODS

### Identification of eligible studies and data extraction

A literature search was performed in the databases of PubMed and Embase to identify studies through December 8, 2016. The following words were used: “tumor necrosis factor alpha inducible protein 3,” ‘‘glaucoma,’’ ‘‘primary open angle glaucoma,’’ ‘‘POAG,’’ ‘‘primary angle closure glaucoma,’’ ‘‘polymorphism,’’ ‘‘PACG’’ “cyclin-dependent kinase Inhibitor-2B” and ‘‘CDKN2B’’. No restrictions were placed on the search. Hand screening was conducted in order to identify additional studies. The identified studies conformed to the following criteria: (1) studies that evaluated the association between glaucoma and CDKN2B gene rs1063192 polymorphism, (2) studied on human beings, (3) study provided sufficient data to calculate the odds ratios (ORs) and 95% confidence intervals (CIs), and *P value*, and (4) case-control study.

The relevant data were extracted from the original articles. The extracted information from all eligible studies including: name of first author, publication year, ethnicity, and genotype numbers of cases and controls. Two reviewers independently performed the extraction of data and assessed the study quality based on the NOS [30]. Any discrepancies between the reviewers were resolved by consensus or by a third reviewer.

### Statistical analysis

Statistical manipulations were performed using the Stata 11.0 software (StataCorp, College Station, TX, USA). ORs and 95%CIs were used to assess the strength of associations between CDKN2B gene rs1063192 polymorphism and glaucoma risk. Stratification analysis was carried out by ethnicity, type of glaucoma and source of controls. Cochran's Q statistic was also used to assess within- and between study variation, and tests of heterogeneity were used to assess the null hypothesis that all studies evaluated the same effect. When a Q test indicated *P* < 0.1 or I^2^ > 50% indicated heterogeneity across studies, a random-effect model was used. Otherwise, the fixed-effects model was applied [31]. Pooled ORs were calculated for allele model, dominant model, recessive model, homozygous model, and heterozygous model. Sensitivity analyses were performed by omitting each study in turn to determine the effect on the test of heterogeneity and evaluated the stability of the overall results. We assessed the departure from the HWE in the controls using Pearson's χ2 test. Potential publication bias was assessed by Begger's and Egger's linear regression test [32]; *P* < 0.05 was considered to indicate statistically significant. The power of this meta-analysis was calculated with a significant value of 0.05 [33].

## Abbreviations

CDKN2B: cyclin-dependent kinase Inhibitor-2B
POAG: primary open-angle glaucoma
PACG: primary angle closure glaucoma
IOP: intraocular pressure
CI: confidence interval
OR: odds ratio
NOS: Newcastle-Ottawa Scale
SNP: single nucleotide polymorphism
GWAS: Genome-wide association studies
